# A Comprehensive Study of Historical Detection Data for Pathogen Isolates from U.S. Cattle

**DOI:** 10.3390/antibiotics12101509

**Published:** 2023-10-03

**Authors:** George Gu, Henry Pei, Alan Zhou, Brianna Fan, Hanlin Zhou, Austin Choi, Zuyi Huang

**Affiliations:** Department of Chemical and Biological Engineering, Villanova University, Villanova, PA 19085, USA; georgegu2006@gmail.com (G.G.); peihenryi@gmail.com (H.P.); alanzhou1845@gmail.com (A.Z.); briannafan25@gmail.com (B.F.); hanlinz3128@gmail.com (H.Z.); acaustinchoi@gmail.com (A.C.)

**Keywords:** foodborne pathogens, antimicrobial resistance, cattle, principal component analysis, data analysis

## Abstract

Foodborne pathogens pose substantial health hazards and result in considerable economic losses in the U.S. Fortunately, the National Center for Biotechnology Information Pathogen Detection Isolates Browser (NPDIB) provides valuable access to antimicrobial resistance (AMR) genes and antimicrobial assay data. This study aimed to conduct the first comprehensive investigation of AMR genes in pathogens isolated from U.S. cattle over the past decade, driven by the urgent need to address the dangers of AMR specifically originating in pathogens isolated from U.S. cattle. In this study, around 28,000 pathogen isolate samples were extracted from the NPDIB and then analyzed using multivariate statistical methods, mainly principal component analysis (PCA) and hierarchical clustering (H-clustering). These approaches were necessary due to the high dimensions of the raw data. Specifically, PCA was utilized to reduce the dimensions of the data, converting it to a two-dimensional space, and H-clustering was used to better identify the differences among data points. The findings from this work highlighted *Salmonella enterica* and *Escherichia coli* as the predominant pathogens among the isolates, with *E. coli* being the more concerning pathogen due to its increasing prevalence in recent years. Moreover, tetracycline was observed as the most commonly resistant antimicrobial, with the resistance genes *mdsA*, *mdsB*, *mdtM*, *blaEC*, and *acrF* being the most prevalent in pathogen isolates from U.S. cattle. The occurrence of *mdtM*, *blaEC*, *acrF*, and *glpT_E448k* showed an increase in pathogens isolated from U.S. cattle in recent years. Furthermore, based on the data collected for the locations of AMR cases, Texas, California, and Nebraska were the major areas carrying major AMR genes or antimicrobials with detected resistance. The results from this study provide potential directions for targeted interventions to mitigate pathogens’ antimicrobial resistance in U.S. cattle.

## 1. Introduction

Foodborne pathogens originating from cattle are a prevalent issue that result in massive economic loss, as well as various diseases worldwide [1]. These foodborne pathogens can be spread to humans through improperly cooked food, including meat and unpasteurized dairy products [2,3]. Nationally, it is estimated that foodborne illnesses in humans cause a loss of $90 billion and result in 128,000 hospitalizations and 3000 deaths annually [4]. The total number of cattle and calves in the U.S., as of 1 January 2023, amounted to 89.3 million heads, with 2.83 million heads of cattle slaughtered in 2022 to produce 2.33 billion pounds of beef [5]. Cattle meat can become contaminated with foodborne pathogens at various stages of the meat production process, from farm to fork. For example, cattle can carry foodborne pathogens in their gastrointestinal tract. There exists a potential for fecal contamination to encounter the meat during the slaughtering process [6]. In addition, certain cattle, often referred to as “super shedders,” have been documented as exerting a significant effect on the prevalence and transmission of *E. coli* O157:H7 in the environment [7]. In 2007 and 2008, *Escherichia coli* O157:H7 was identified as the cause of the 40 million pounds of beef that were recalled for possible contamination [8]. This is because *E. coli* O157:H7 is known to cause hemorrhagic colitis and hemorrhagic uremic syndrome in humans [9], which pose a major health concern. In another study conducted on *Salmonella enterica* Serotype Cerro, one of the foodborne pathogens commonly originating from cattle, it was found that the prevalence of *Salmonella enterica* Cerro was growing in cattle in New York. This poses a significant threat to both human health and the health of livestock [10].

Due to the prevalence of pathogens present inside of agricultural and food production environments, antimicrobials have been widely used to combat these pathogens. However, foodborne pathogens are developing resistance to commonly used antimicrobials, not only fostering cross resistance in human medicine, but affecting the safety of consumer goods as well [11]. For example, the resistance of *Salmonella* Typhimurium to tetracyclines increased from 0% to 98% in a span of 50 years [12]. It was found in a study conducted on *Klebsiella pneumoniae* that all strains possessed multidrug resistance to several clinical antimicrobials [13]. Another study showed that there was an increase in the resistance of the three major Bovine Respiratory Disease (BRD) pathogens to most antimicrobials commonly used for the treatment of BRDs [14]. The AMR pathogens can be transmitted from cattle to humans. This makes the treatment of foodborne illness far more challenging [15]. In one study, the use of aminoglycosides as an antimicrobial in livestock was found to increase the selective pressure on *E. coli* for the development of antimicrobial resistance, which could go on to infect humans [16].

AMR is conferred by resistance genes encoding proteins that render antimicrobials ineffective. A study performed on the foodborne pathogen *Streptococcus agalactiae* showed that there was a high prevalence of AMR genes. Some of the AMR genes found in high frequencies include *tetM*, *ermB*, and *tetO*. Among these genes, *TetM* and *tetO* are genes that encode tetracycline resistance in bacteria, while *ermB* is a gene that encodes the ribosomal methylase, which causes resistance to macrolides. These genes were found to be essential for the sustainability of AMR resistance in these pathogens [17]. Additionally, *Campylobacter* isolates were detected in a study with the presence of several AMR genes conferring resistance to tetracyclines and beta-lactams, among other antimicrobials [18]. In cattle, *Bacteroides fragilis* and *Enterobacter aerogenes* were found to be the hosts of several genes, such as the transporter gene and regulator; efflux pump; and genes that are involved in the resistance to polymyxin, aminoglycoside, beta-lactam, and macrolides [19]. The horizontal transfer of AMR genes is one of the main driving forces for the spread and prevalence of antimicrobial resistance in foodborne pathogens. The transfer of AMR genes can occur in all stages of the food production chain [20]. For example, a study discovered a total of 97 AMR genes in animal manure, wastewater, and drinking water [21]. When these cattle foodborne pathogens with AMR genes infect humans, the illnesses they cause become difficult to treat using traditional antimicrobials [16]. Therefore, the study of AMR genes found in the foodborne pathogens infecting cattle is vital to address the issue of the AMR of foodborne pathogens infecting humans [10].

Obtaining AMR gene and resistance data over time in different locations is essential to provide a comprehensive view of AMR status in U.S. cattle. Fortunately, the NCBI Pathogen Detection Isolates Browser (NPDIB) provides the genotype data of pathogen isolates [22,23,24]. Additional information on pathogen isolates, such as the sampling location, the sampling date, and isolation sources, is also included. The value of the NPDIB in studying antimicrobial resistance has been demonstrated by several studies (refer to [25,26,27] for examples). However, none of these existing studies were focused on the AMR status of pathogens isolated from U.S. cattle. On the other hand, cattle foodborne pathogens with acquired AMR are urgent issues due to the serious economic and health concerns. Even though a significant amount of data on U.S. cattle is present in the NPDIB database, little work has been performed to provide a systematic and spatiotemporal analysis of pathogens and AMR genes for U.S. cattle.

This study analyzed the AMR genotype and antimicrobial susceptibility testing data for 28,000 pathogen isolates from the NPDIB for U.S. cattle during 2013–2023 to determine the extent of and spatiotemporal trends in antimicrobial resistance in U.S. cattle. The major pathogen, AMR genes, and antimicrobials involved in antimicrobial resistance cases in U.S. cattle were thoroughly studied from the data. Because of the large number of pathogen samples and the presence of high-dimensional variables in the data, multivariate statistical analysis methods were implemented in the analysis. In particular, principal component analysis (PCA) [28,29,30,31] was used to reduce the data into a two-dimensional space that is useful for visualizing pathogens, AMR genes, and antimicrobials with detected resistance. Based on the projection of data on the two-dimensional space, hierarchical clustering [32,33,34] was further implemented to study the relationships and similarities shown in those pathogens, genes, or antimicrobials. Finally, the occurrence frequencies of antimicrobial resistance cases were projected onto the U.S. map and then presented in time charts to study the spatiotemporal trends of the AMR status and cross-validate the findings from multivariate statistical analysis. This study thus provides a comprehensive view of antimicrobial resistance in U.S. cattle, which is essential to understand and take initiative in preventing foodborne pathogens from developing further antimicrobial resistance. Additionally, the approach used in this study can be easily applied to other types of livestock, such as poultry or swine, to understand antimicrobial resistance and AMR genes in foodborne pathogens originating from these animals that can be targeted with future antimicrobials.

## 2. Results

### 2.1. Analysis of Pathogens Isolated from U.S. Cattle

**Figure 1:**
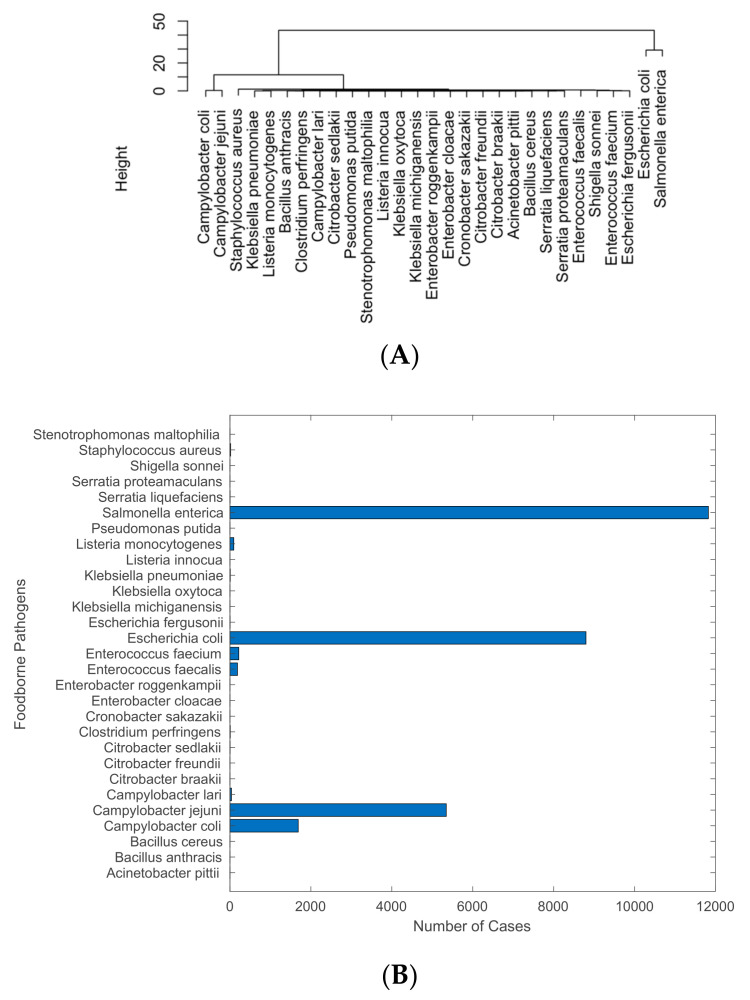
(**A**) The H-clustering result of the foodborne pathogens to show the similarity of their occurrences. (**B**) The bar plot showing the case numbers for foodborne pathogens isolated from U.S. cattle.

**Figure 2:**
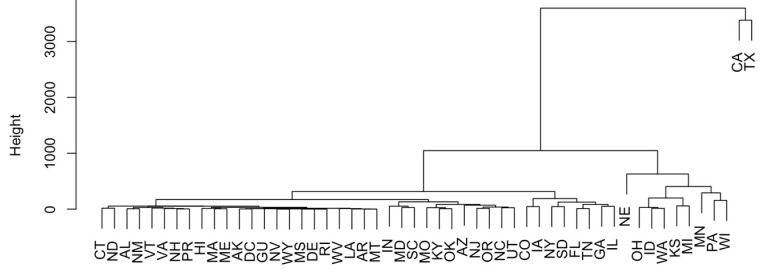
The H-clustering of U.S. states to show the similarity of the occurrence of AMR genes in them.

While the PCA was conducted to identify the outlier pathogens, quite a few nonoutlier pathogens were clustered together (Supplementary Materials Figure S1). *Salmonella enterica* and *E. coli* are the major outliers, with *Campylobacter jejuni* and *Campylobacter coli* also varying from the clustered group. On the dendrogram in Figure 1A, *Salmonella enterica* and *E. coli* are far apart from the other points, with *Campylobacter coli* and *Campylobacter jejuni* also being significantly farther than the others. While certain pathogens are clustered in the PCA figure (Figure S1), the dendrogram (Figure 1A) can further uncover those pathogens. Figure 2B details the number of total cases for the pathogens, and the results show that the two most significant and prevalent pathogens include *Salmonella enterica* (~12,000 cases) and *E. coli* (~9000 cases). This is consistent with results from the PCA and H-clustering. Additionally, *Campylobacter jejuni* and *Campylobacter coli* both stand out in Figure 2A,B.

PCA and H-clustering were conducted on the number of cases for each state to study the similarity of these states according to the pathogens detected in them. Principal component analysis shows that California and Texas are significantly greater in distance from the rest of the data points (Figure S2). There are also some other points that vary from the norm, such as Nebraska, Wisconsin, and Pennsylvania, but not as much as California and Texas. In the dendrogram in Figure 2, California and Texas are separated from the rest of the points. Nebraska, Wisconsin, Pennsylvania, Minnesota, Michigan, Kansas, and Oklahoma also stand out in Figure 2.

**Figure 3:**
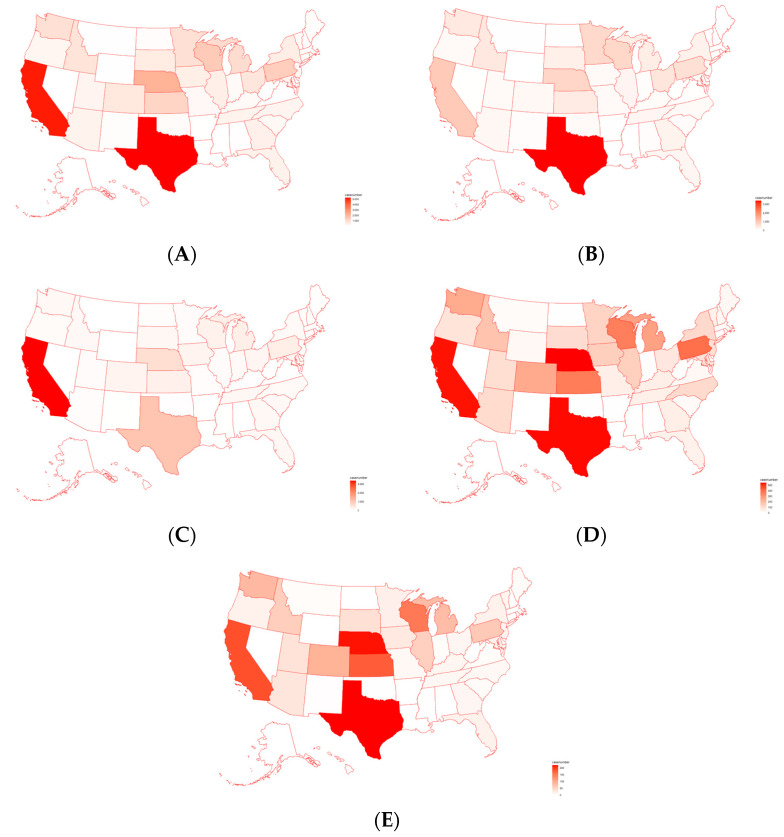
(**A**) Total cases of foodborne pathogens detected in individual states on a U.S. map; (**B**) *Salmonella enterica* cases detected in individual states on a U.S. map; (**C**) *Escherichia coli* cases detected in individual states on a U.S. map; (**D**) *Campylobacter jejuni* cases detected in individual states on a U.S. map; (**E**) *Campylobacter coli* cases detected in individual states on a U.S. map.

The number of total pathogen cases was plotted on a U.S. map to cross validate the results derived from PCA and clustering. As shown in Figure 3A, the detected cases of pathogens were significantly greater in the states of California and Texas (~5000 cases each), as shown by the dark red highlights depicted on the map. In addition, the states of Pennsylvania, Nebraska, and Wisconsin were also found to have thousands of cases each (~2000–3000), as illustrated by the lighter red highlights on the map. Maps of individual foodborne pathogens in the U.S. were also generated in Figure 3B–E, individually for the top four pathogens (*Salmonella enterica*, *E. coli*, *C. jejuni*, *C. coli*). *Salmonella enterica* was found to have a significant number of cases in Texas, while *Escherichia coli* had the majority of its cases in California. Other states, such as Nebraska, Wisconsin, and Pennsylvania, also had a moderate number of cases for all four pathogens. Therefore, the results from PCA and clustering were supported through the plotting of pathogen cases on a U.S. map, showing that pathogen cases are most prevalent in California and Texas.

**Figure 4:**
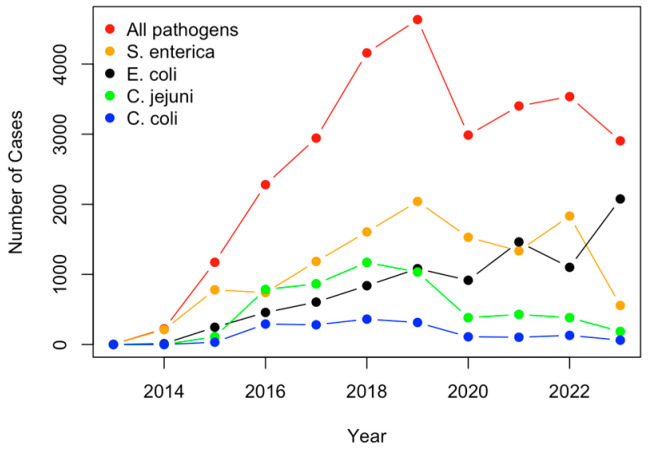
The number of pathogen cases plotted over time for the top four pathogens identified via PCA and H-clustering (note: data for 2023 is up to 15 June 2023).

The profiles of occurrence cases depicting the time trends (2013–2023) for the top four pathogens with the largest number of total recorded cases are shown in Figure 4. Overall, it was observed that the total number of pathogen cases was on a steady increase until it spiked in 2019, where it encountered a sharp decline in 2020, as well as a small increase shortly afterward. As observed from Figure 4, *Salmonella enterica* cases (orange) experienced a sharp decline in the last two years, while *Escherichia coli* cases (black) have greatly increased. The rise in *Escherichia coli* cases may pose a risk to human health, and this should be further researched.

### 2.2. Analysis of Antimicrobials with Detected Resistance in Pathogen Isolates from U.S. Cattle

**Figure 5:**
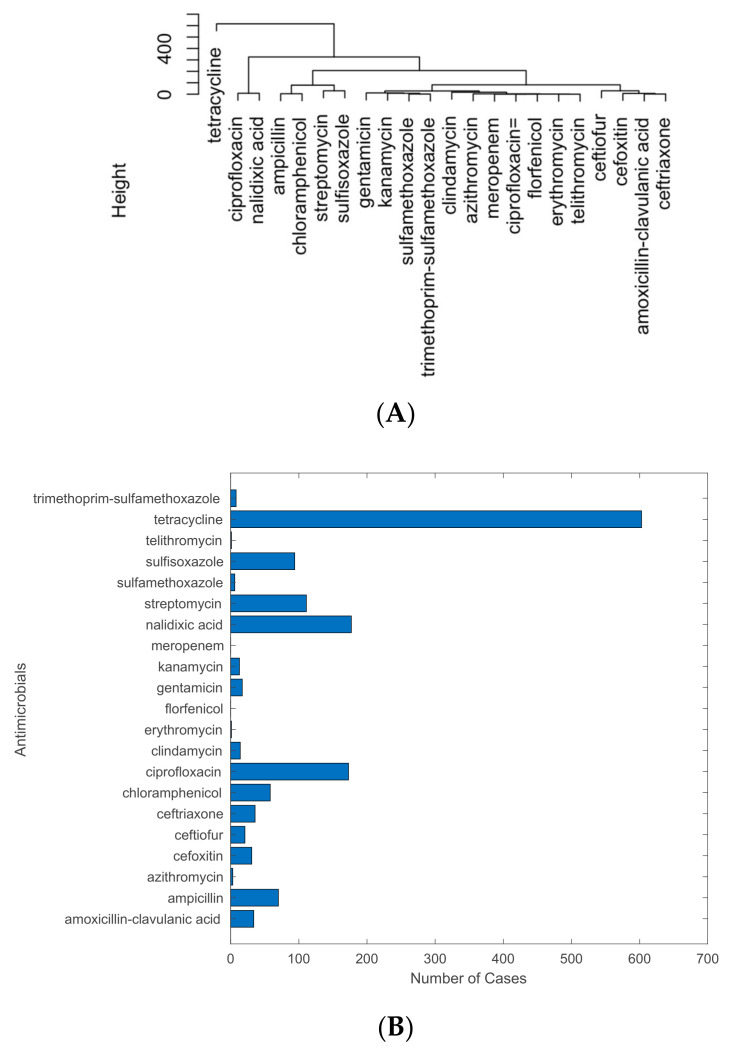
(**A**) H-clustering performed for the top antimicrobials that pathogens resist in U.S. cattle. (**B**) A bar graph showing the number of samples with detected resistance to each antimicrobial.

Principal component analysis and hierarchical clustering were employed to analyze the antimicrobials resisted by pathogen isolates in U.S. cattle. While certain antimicrobials are clustered in the PCA figure (Figure S3), they are separated in the H-clustering results (Figure 5A). The results emphasize tetracycline as the most resisted antimicrobial among the detected pathogens in cattle. The other most resisted antimicrobials include ciprofloxacin, nalidixic acid, ampicillin, chloramphenicol, streptomycin, and sulfisoxazole. These antimicrobials are separated from others in both PCA and H-clustering analysis. These results are further supported by the bar chart (Figure 5B), with tetracycline having the greatest number of pathogen isolates with resistance to it, and the other six having the six highest frequencies of resistance. Among the top seven antimicrobials, nalidixic acid and sulfisoxazole are veterinary drugs used to treat urinary tract infections (UTIs). UTIs in cattle can be caused by many common pathogens, such as *E*. *coli* and *Staphylococcus.* The pathogens that are exposed to these antimicrobials may generate resistance. Ciprofloxacin is one of the most commonly used antimicrobial agents for bacterial infections in livestock [35]. This may cause significant exposure of this antimicrobial to pathogens and thus increase the likelihood of developing resistance.

**Figure 6:**
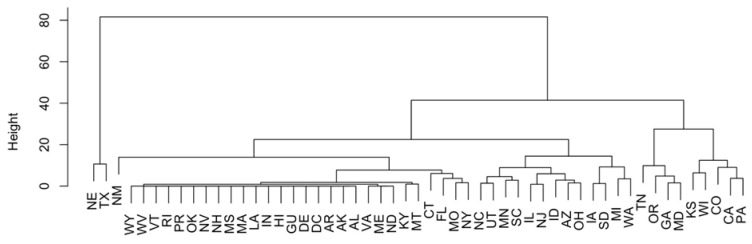
H-clustering performed to find states that had cattle that were most resistant to antimicrobials.

**Figure 7:**
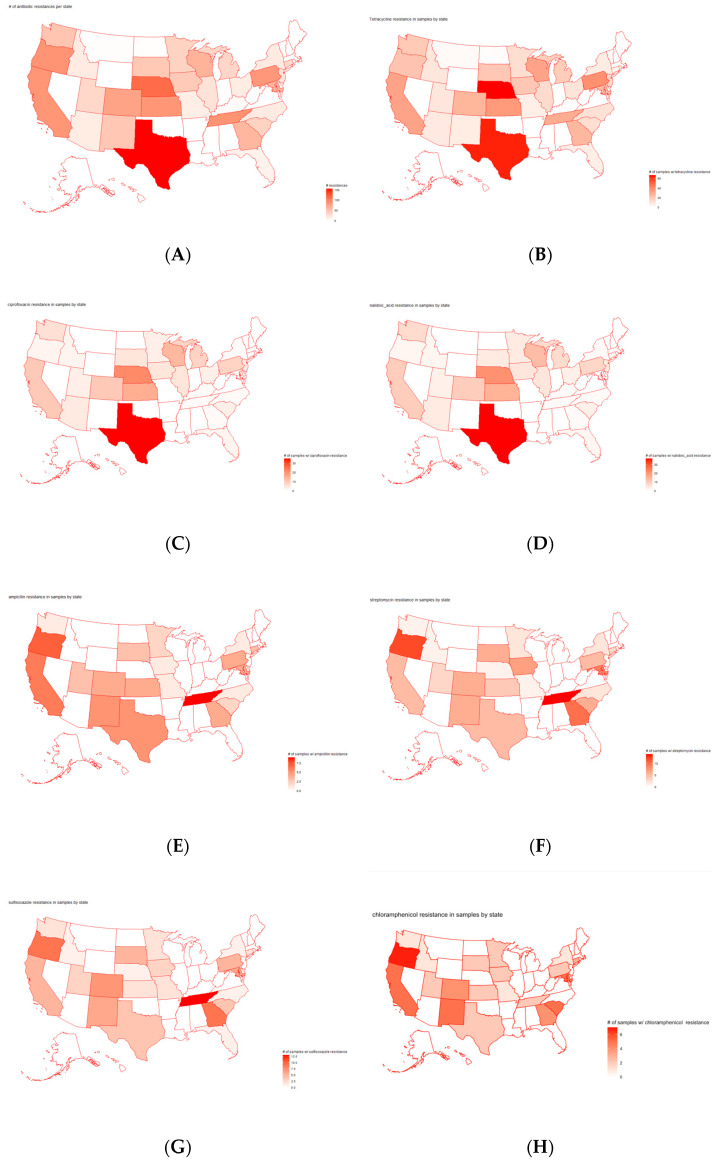
The U.S. map of the number of cases with detected resistance to (**A**) all antimicrobials, (**B**) tetracycline, (**C**) ciprofloxacin, (**D**) nalidixic acid, (**E**) ampicillin, (**F**) streptomycin, (**G**) sulfisoxazole, and (**H**) chloramphenicol.

Principal component analysis and H-clustering were implemented to study the states for pathogens isolated from cattle that were the most resistant to certain antimicrobials. The results (Figure S3 for PCA and Figure 6 for clustering) show that states, like Texas and Nebraska, have the highest number of cases with detected resistance to certain antimicrobials. To verify these results, a U.S. map that plots the number of cases with antimicrobials resisted by pathogens per state was generated (Figure 7A). The graph reflects the results of the PCA and H-clustering, with Texas and Nebraska being the two states with the darkest redness. Additionally, maps were created for the top seven antimicrobials found previously. Figure 7B–H show the number of samples for each antimicrobial with detected resistance for each state. These maps showed that samples with resistances to tetracycline, ciprofloxacin, and nalidixic acid were most frequently found in Texas and Nebraska. For the other four antimicrobials, Tennessee and Oregon were the largest hotspots for resistance, except for chloramphenicol, which had the most cases in Oregon and California.

**Figure 8:**
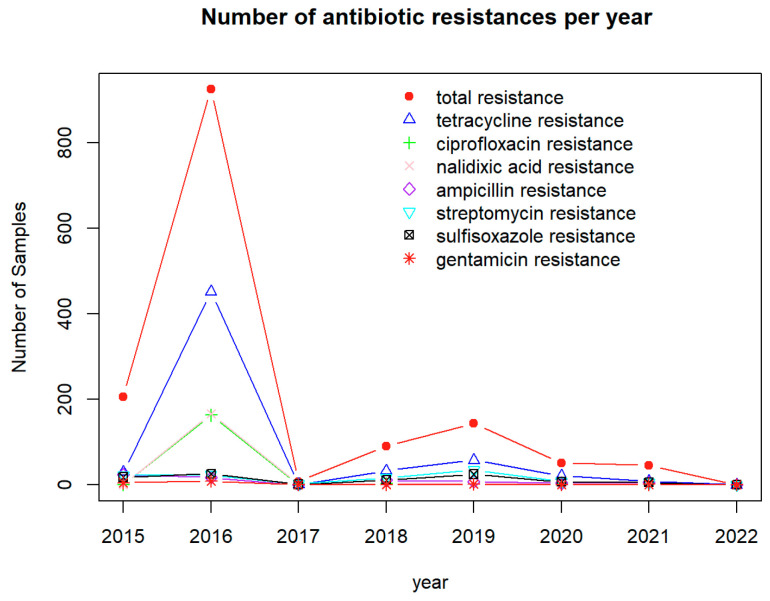
The time profile indicating the number of samples with detected resistance to the top antimicrobials for each year in the dataset.

Figure 8 shows the time profile for the number of cases with detected resistance to the top antimicrobials identified via PCA and H-clustering. The overall trend shows that resistance to antimicrobials detected in foodborne pathogens spiked in 2016, with a smaller peak in 2019. Furthermore, the large spike in 2016 was observed to be true for all the antimicrobials, but the peak in 2019 was only seen for four out of the seven antimicrobials, including tetracycline, streptomycin, chloramphenicol, and sulfisoxazole.

### 2.3. Analysis of Antimicrobial Resistance Genes in Pathogens Isolated from U.S. Cattle

**Figure 9:**
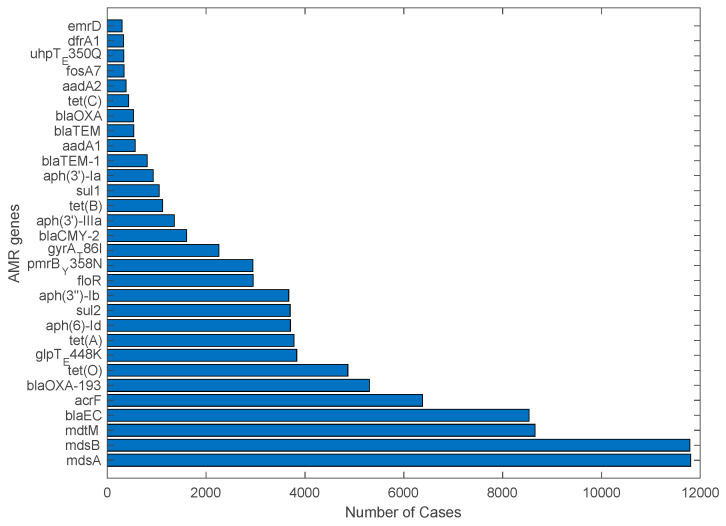
The 30 genes with the largest detected cases in the dataset.

It was observed from the principal component analysis that *mdsA*, *mdsB*, *mdtM*, *blaEC*, *acrf*, and *glpT_448K* emerged as the most prominent outliers (Figure S5). Since there are more than 300 genes in the dataset, it was extremely difficult to show all genes clearly in a dendrogram (not shown). The solution to this is to use “cut the tree” function in R, which partitions the dendrogram into various numbers of clusters (i.e., two clusters and then three clusters and so on). The genes from different clusters were compared to the outlier genes shown in the zoomed-in PCA figure. Due to the space constraint, only the top 30 significant genes were further studied. In particular, a bar plot was generated (Figure 9) displaying the actual number of occurrences of each of these genes in the dataset. This plot further cross-validated the results from PCA and H-clustering. For example, genes *mdsA* and *mdsB* were the top two in terms of the number of detected cases, with approximately both having around 12,000 cases. These genes, along with the other genes in the top 30, provide a good start for future study in order to brainstorm countermeasures against AMR pathogens.

While the top 30 genes were all statistically relevant, for the purposes of this study, more focus was put into analyzing the top 10 genes in order to provide more detail on them. The website UniProt, a freely accessible resource of protein sequences and functional information, was used to annotate the genes. The multidrug efflux RND Resistance-Nodulation-Division (RND) transporter system is a type of protein complex found in bacteria that is involved in the efflux of various drugs and toxic compounds from the cell. Multidrug Sensitivity protein A (*mdsA*) and *mdsB* are components of this transporter system commonly found in *Salmonella enterica* [36] explaining why they are the top one and two as *S. enterica* was one of the leading pathogens in terms of case numbers from the dataset. The multidrug resistance protein *mdtM* is a type of membrane transporter found in bacteria that is involved in multidrug resistance and is also present in both *S. enterica* and *E. coli* [37] (the top two pathogens identified in this study). Beta-lactamase (*blaEC*) is an enzyme produced by certain bacteria (e.g., *E. coli*) that confers resistance to beta-lactam antibiotics [38], such as penicillins, cephalosporins, and carbapenems. The gene *AcrF* is an efflux pump membrane transporter that plays a crucial role in the efflux of molecules, including drugs, toxins, and metabolites, out of cells, also common in *E. coli* [39]. The gene *BlaOXA-193* is part of class D beta-lactamases commonly known for their ability to hydrolyze a broad spectrum of beta-lactam antibiotics, including penicillins and cephalosporins, rather than transport them out of bacteria [40]. The gene *BlaOXA* was only found in one result in UniProt in the *C. jejuni* pathogen, which was the third most common pathogen, which makes sense why it is not higher on the list. *glpt_E448K* was found in 90% of *E. coli* from clinical and environmental samples [41]. The prevalence of fosfomycin resistance in *E. coli* isolated from cattle was found to be driven by a point mutation in *glpT_E448K* [42]. While *tet*(*A*) and *tet*(*O*) are both common tetracycline resistance genes, *tet*(*A*) and *tet*(*O*) are mainly in gram-negative bacteria and the gram-positive bacteria, respectively [43]. The gene *aph(6)-Id* is a bona fide streptomycin phosphotransferase that confers resistance to streptomycin in *E. coli* [44].

**Figure 10:**
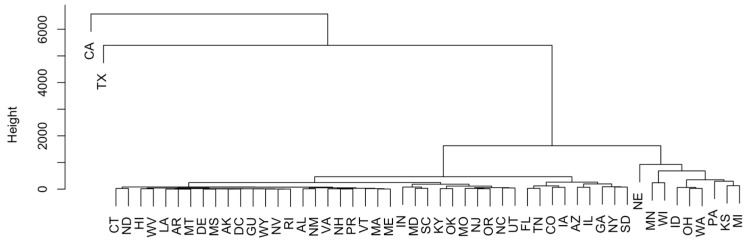
H-clustering analysis to show the similarity of individual states for the AMR genes they carry.

**Figure 11:**
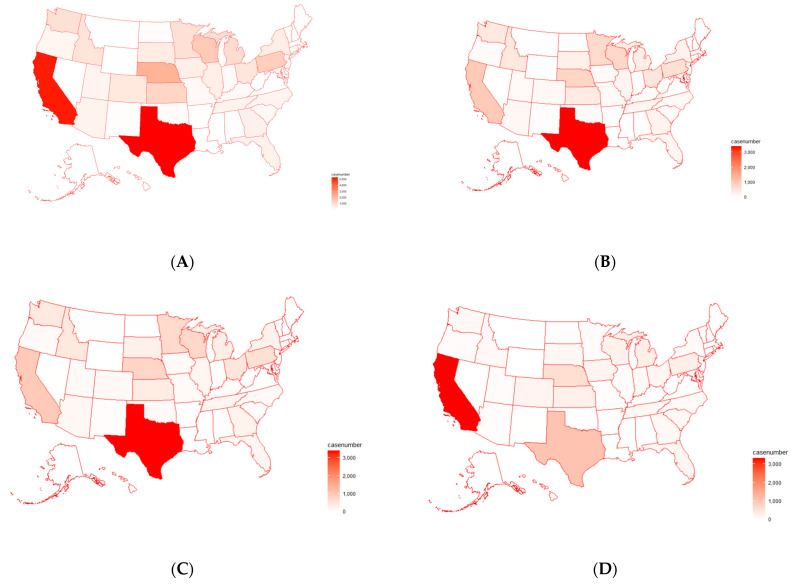

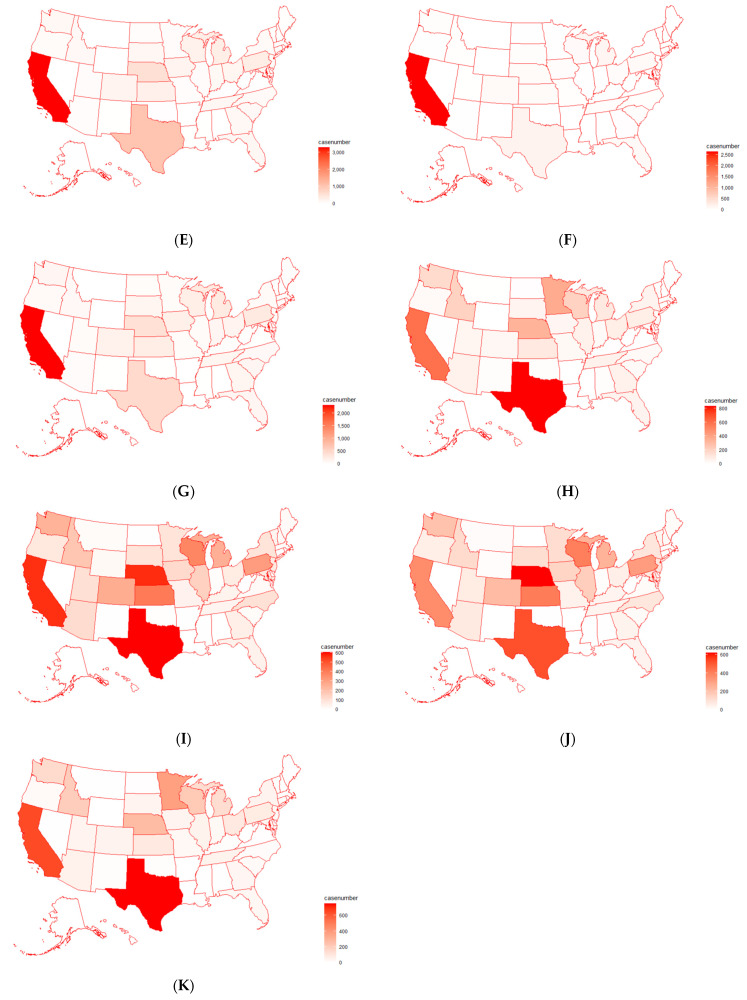
(**A**) The U.S. map indicating the detected case numbers for all of the top 30 AMR genes in individual states; the U.S. map indicating the detected case numbers in individual states for (**B**) *mdsA*, (**C**) *mdsB*, (**D**) *mdtM*, (**E**) *blaEC*, (**F**) *glpT_E448K*, (**G**) *acrF*, (**H**) *tet(A)*, (**I**) *blaOXA-193*, (**J**) *tet(O)*, and (**K**) *aph(6)-Id*.

Following the steps taken to analyze the data for AMR genes and pathogens, principal component analysis (Figure S6) and H-clustering (Figure 10) were conducted on individual U.S. states for the detected AMR genes. It was observed that Texas and California appeared as the most obvious outliers in both the PCA and the H-clustering dendrogram. Following California and Texas, Minnesota, Nebraska, Wisconsin, and Pennsylvania carried a high number of AMR genes, although far fewer than that of either California or Texas. The rest of the states were far less significant in general. Cross-validation of the results from this PCA and H-clustering was conducted based on the U.S. map indicating the detected cases of all AMR genes in each state (Figure 11A) and separate U.S. maps for each of the top 10 AMR genes in each state (Figure 11B–K). The result was that California and Texas had the most cases by far on the total map and most commonly occurred in the other 10 maps. It is worth noting that while *mdsA* and *mdsB* were almost only located in Texas, *mdtM*, *blaEC*, *acrF*, and *glpT_E448K* were primarily located in California. The rest of the top 10 AMR genes were more spread across the map, although all of them appeared in at least one of the two prominent states, California or Texas.

**Figure 12:**
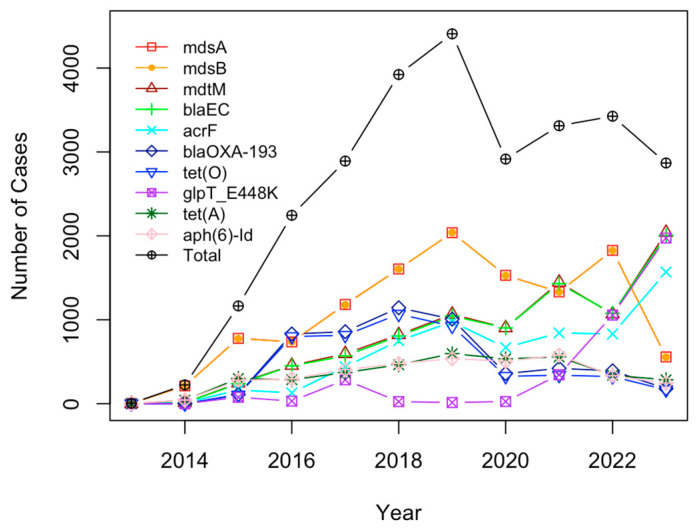
The time profile for the number of samples of the top 10 genes per year (note: data for 2023 is up to 15 June 2023).

Finally, a time trend analysis was conducted for the top 10 genes (shown in Figure 12). The results showed a slow start in 2014 leading to a period of steep growth, eventually dropping somewhat, starting from 2019, but still maintaining around 3000 samples per year. Since the year 2023 has not ended yet, it is not included in the time profile. There were many pairs of genes that had nearly identical time trends, such as *msdA* and *msdB* (the overlaid orange and red lines). The genes that are on the rise in recent years include *mdtM*, *blaEC*, *acrF*, and *glpT_E448K*.

## 3. Materials and Methods

**Figure 13:**
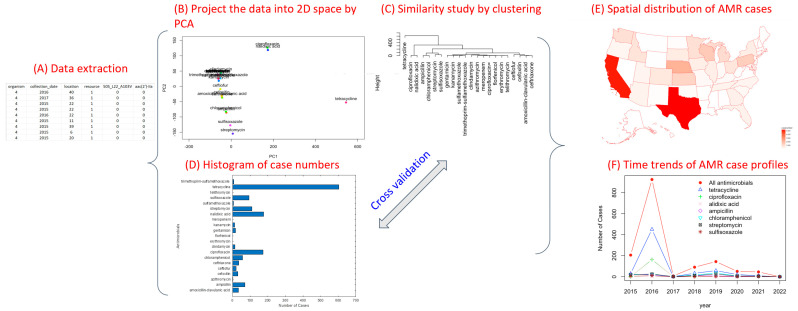
The overview of data extraction and analysis to study antimicrobial resistance of pathogens isolated from U.S. cattle.

While more detail of these approaches will be provided in the following subsections, Figure 13 shows the overview of the approaches designed in this study to extract data from the NPDIB database and conduct data analysis. Specifically, the pathogen isolate data for U.S. cattle, which contain detected AMR genes and antimicrobial susceptibility testing results in different states on different dates, was downloaded. MATLAB programs were developed to extract the data into a matrix (as shown in Figure 13A), in which each row represents a pathogen isolate and each column represents one variable (e.g., pathogen name, sampling time, sampling location, whether an AMR was detected, and whether an antimicrobial was tested with resistance). The high-dimensional space data were then projected via principal component analysis into a two-dimensional space for visualization (Figure 13B). Since certain items (antimicrobials as examples) are clustered together, it is challenging to show all of those items in the figures. However, the value of PCA is that the outliers shown in the PCA projection may indicate their important involvement in antimicrobial resistance. These outliers can be further confirmed through hierarchical clustering as they are generally located in the branch separated from others in the dendrogram tree (Figure 13C). For example, tetracycline resistance is notably distinct from the other antimicrobials in Figure 13B,C. This finding is orthogonally supported by the bar graph, which shows that the detection frequency of tetracycline resistance is disparately larger than that of other antimicrobials (Figure 13D). Spatiotemporal patterns of AMR were further studied through the creation of distribution maps and historical profiles. In particular, the spatial trends in AMR cases were studied by mapping the data points with AMR resistance to the location from where they were collected, creating a graph showing the number of cases per state using a gradient. Figure 13E shows the cases of tetracycline resistance detected in individual states in the U.S. from 2015 to 2023. California and Texas are the top states with the largest number of tetracycline resistance cases. To study the temporal trend in antimicrobial resistance, the occurrence frequency of AMR cases was plotted over the years. Figure 13F shows that the maximum tetracycline resistance cases were detected in 2016. Since the relationship between the studied items can be illustrated by an H-clustering diagram, PCA figures are provided in the appendix for reference.

### 3.1. Materials

Around 28,000 pathogen isolate samples were downloaded from the NPDIB database by filtering the samples that are of AMR genotype data specific to U.S. cattle. Among these samples, only 1500 contain antimicrobial susceptibility testing results. It turns out that the samples with AMR genes were mainly obtained since 2013, and the samples with antimicrobial susceptibility testing were reported since 2015. The samples used in this study are thus mainly for the period of 2013–2023. A MATLAB program was developed to extract and store the data in *.csv files. The dataset was arranged in a matrix format, with each row corresponding to a pathogen sample and each column representing different variables for each sample, including the (1) pathogen type, (2) collection date, (3) location (i.e., states), (4) resource (cattle type), (5) presence of AMR genes, and (6) susceptibility tests for different antimicrobials. Table 1 shows a portion of the data matrix for the purpose of an illustration. The 29 foodborne pathogens were identified and indexed with individual numbers in the matrix. Similarly, the location of each sample collected was also indexed by number, with each number representing a different state or region (in the range of 1–53). The resistances to 22 different antimicrobials and the presence of 311 AMR genes found in each sample are represented by a 1 (found) or a 0 (not found). For example, gene *aac(3)-Via* was detected in the first sample for pathogen *Campylobacter coli* (i.e., organism = 4) in the state of Rhode Island (i.e., location = 40) in 2016. As for the antimicrobial susceptibility testing, this sample of *Campylobacter coli* showed resistance to streptomycin, sulfisoxazole, and tetracycline (indicated by the value of 1 in the first row under the corresponding antimicrobials in Table 1). The data were then imported into RStudio for analysis.

### 3.2. Methods: PCA, H-Clustering, and Spatiotemporal Trend Analysis

Since the dataset contains more than 28,000 samples that can comprise more than 300 variables (e.g., 311 genes and 22 antimicrobials), principal component analysis (PCA) was firstly used to project the data into a two-dimensional space. PCA is known for its capability of reducing multidimensional data into an easily comprehensible two-dimensional format while retaining much of the vital information stored in the data. Before PCA could be utilized, the data were arranged into a two-dimensional matrix. For example, in order to identify the pathogens carrying the most antimicrobial resistance genes, pathogens were placed in rows and the AMR genes were placed in columns, with each value in the matrix representing the number of cases with the pathogen in the row carrying the AMR gene in the column. Similarly, AMR genes were placed in rows and pathogens were placed in columns to identify the most common AMR genes carried by pathogens. In principal component analysis, a new coordinate direction called Principal Component 1 (PC1) was identified with the largest variance in the projections of the data points onto that direction. Similarly, PC2 was identified, and it is orthogonal to PC1. The data points are then projected into the PC1-PC2 space to provide initial indications of group similarity and simplify the process of identifying outliers. These outliers generally indicate unusually high rates of occurrence compared to the other sample.

While PCA can visualize the data in a two-dimensional space, the relationships between data points are not specifically quantified. Some data points can be clustered together. Hierarchical clustering (H-clustering) was thus employed to quantify and better identify the relationships between data points. H-clustering works by first calculating the distance between every two data points from the projections in the PC1-PC2 space and then iteratively linking two data points/sets with the smallest distance between them until all data points are connected in the dendrogram. The data points that are connected through a shorter vertical distance between them indicate that they are more similar in antimicrobial resistance. The H-clustering results do provide an additional avenue to illustrate the similarity of the studied items (e.g., AMR genes, pathogens, antimicrobials with detected resistance, the states in the U.S.) for their involvement of antimicrobial resistance detected in U.S. cattle. The outliers shown in the PC1~PC2 space can be further identified or validated via H-clustering, as they are generally separated from others in the dendrogram. The results from PCA and H-clustering were cross-validated based on bar graphs, which indicate the occurrence frequencies of antimicrobial resistance cases. For example, the important AMR genes identified from principal component analysis and H-clustering generally have higher detection frequencies.

Spatiotemporal patterns of antimicrobial resistance were studied through the creation of distribution maps and historical profiles. The USMAP package from R was integrated with the occurrence data to facilitate the construction of choropleth maps of antimicrobial resistance cases in different states of the U.S. The ggplot2 package in R was used to enhance data visualization in the form of maps [45]. Additionally, the scales package in R was called to apply color gradients to maps [46]. The time trends between the different studied items (e.g., AMR genes, pathogens, antimicrobials, states) were studied by plotting the occurrence frequency profiles over the years. The choropleth maps for individual states and the profiles of AMR occurrence frequencies over the years provide a neat visualization of the antimicrobial resistance status for U.S. cattle across the years and locations in the datasets.

## 4. Discussion

### 4.1. Pathogens Mostly Detected from U.S. Cattle

*Salmonella enterica* and *E. coli* were the pathogens carrying most AMR genes, as indicated by both the PCA, H-Clustering, and bar plot, followed by *C. jejuni* and *C. coli*. This coincides with many previous research studies highlighting the prevalence and dangers of *E. coli*, *Salmonella enterica*, and the other top pathogens found in the results [2,25,47]. A study that analyzed pathogen samples from cattle found that 73% of *E. coli* O157 samples and 77% of *Salmonella* samples possessed multidrug resistance [4]. These findings support the data derived from PCA and H-clustering. Consequently, these top four pathogens are studied more due to the prevalence of AMR and should continue to be studied. As shown by the PCA and H-clustering for pathogen cases by state, California and Texas are clear outliers. Results align with the choropleth maps, which show that *S. enterica* and *E. coli* predominantly inhabit Texas and California, respectively. Since these two pathogens are particularly prevalent, it makes sense that the total map would bias these two states. A possible reason for this bias could be Texas having the largest beef cow industry and California possessing the largest milk cow industry. Finally, analyzing the time chart for the top four pathogens, it seems that although *Salmonella enterica* has been prominent for the last few years, it is now declining in prevalence in U.S. cattle. However, the prevalence of *E. coli* has been increasing in U.S. cattle in recent years [48]. A focus should be placed specifically on *E. coli* due to its increasing prevalence for further research.

### 4.2. Antimicrobials with the Most Detected Resistance by Pathogens Isolated from U.S. Cattle

It was found from this study that the pathogen resistance to tetracycline in cattle is stronger than that to other antimicrobials, as indicated by PCA, H-clustering, and the bar plot for occurrence frequencies. The results fit in with past studies that found high rates of tetracycline resistance in pathogen samples from cattle [49]. Tetracycline administration is highly prevalent in the cattle industry as it is the most common antibiotic used to treat bacterial infections and is also used as a growth promoter. Elevated tetracycline use may explain the distinct resistance profile for this antibiotic relative to the others [50]. The PCA and H-clustering indicate that the highest frequency of detected resistance to antimicrobials was concentrated in states, such as Texas, Nebraska, and Kansas. These findings can be observed in the U.S. map of overall cases of antimicrobials with detected resistance, as well as in the maps of tetracycline. A possible explanation for the large number of samples with AMR would be the high density of feedlot production in these top three states [51]. Feedlots are areas where bacteria, such as *E. coli O157*, are found in over half the water samples in states, such as Kansas and Nebraska [6]. While California was in the top two highest-ranked states for cattle carrying AMR genes and pathogens, the state did not appear in the top three for detected resistance to antimicrobials. A potential reason for this is a 2018 policy enacted by the California Department of Food and Agriculture, which prohibited the preventative use of antimicrobials [52], unless approved by a veterinarian. This measure may reduce the exposure of pathogens to antimicrobials. Although there may be a significant number of pathogens in California and many may carry resistance genes, there is no further selective pressure to become resistant to antimicrobials. Lastly, the time profile showed a significant peak in 2015–2016 for the cases with detected resistance to antimicrobials, with resistance cases considerably dropping, never reaching even one-fourth of the peak. A likely explanation for this spike is that for the years in the smaller antimicrobial data set, the year 2015 had the highest domestic sales of antimicrobial drugs for livestock [53]. The high volume of these sales implied more pathogens were exposed to antimicrobials compared to in other years.

### 4.3. Antimicrobial Resistance Genes Mostly Detected in Pathogen Isolates from U.S. Cattle

The genes *mdsA*, *mdsB*, and *mdtM* were top three most common genes from this study. Based on the research performed in an investigation of stress response genes in AMR pathogens from five countries, *mdsB* and *mdtM* were found to be AMR genes linked to stress response genes [23]. Furthermore, an investigation of the genes involved in the outbreaks of *Escherichia coli* and *Salmonella* spp. in the United States revealed *mdsA* and *mdsB* as AMR genes found in *Salmonella* spp. [24]. Additionally, a study conducted on *E. coli* in cattle found that 78% of the *E. coli* isolates were multidrug-resistant (MDR), testing positive for resistance to beta-lactams (*blaCTX-M*), and *tet(A)* [49]. This is consistent with the results from this study in which *E. coli* was a major carrier of AMR genes and the genes *blaCTX-M* and *tet*(*A*) were among those genes with the highest detected occurrences. Studying the U.S. map for AMR gene detection (i.e., Figure 11), Texas and California had by far the most cases of AMR genes in pathogens. This makes sense because the expansion of dairy farms in the U.S. to hotter climates, such as southern Texas and California, has come with the problems of heat stress and cow comfort. Stress and variations in the stage of milk production may render cattle more susceptible to bacteria, thus leading to an obvious trend in the data [47]. From the timeline trends of the top 10 AMR genes, some of them were decreasing in recent years, but four of the top 10 (*mdtM, blaEC, acrF,* and *glpT_E448k*) showed an increase in recent years, which is concerning. The genes that have exhibited increased prevalence in recent years are prevalent in *E. coli*. This underscores the growing concern regarding the rise in *E. coli*, particularly since all other pathogens have been declining in recent years. Future research should be directed at trying to stop the spread of these AMR genes or coming up with new antibiotics or other countermeasures against these AMR genes within pathogens.

### 4.4. Limitation and Future Work

While multivariate statistical analysis approaches were implemented to uncover antimicrobial resistance patterns hidden in the NPDIB database, some limitations exist in this study. First, the data from the NPDIB database may have been limited due to the struggle of collecting data during the COVID-19 pandemic. As a result, the data analyzed throughout this study may not be as accurate as it could have been if the pandemic had not occurred. Additionally, pathogen and AMR gene analysis included data from 2023, which may be incomplete due to the time of year. The data for 2023 in the time profiles (i.e., Figure 4, Figure 8 and Figure 12) were not complete either. While there are around 28,000 samples containing information for AMR genes in pathogens isolated from U.S. cattle, only 1400 samples come with antimicrobial susceptibility testing. More samples with antimicrobial susceptibility testing would be helpful to study the cases of antimicrobials with detected resistance.

While this research represents the inaugural in-depth exploration of antimicrobial resistance in pathogens isolated from U.S. cattle using data sourced from the NPDIB database, there remains ample scope for further investigation to address lingering questions and hypotheses. One example of a potential area of study would be the difference between young and old cattle. The comparison of data collected for young and old cows may indicate the spread of AMR genes over the growth of cattle. Comparisons can also be conducted for female and male cattle and for (milk) cows and (meat) beef. In addition, recent advances in computational techniques, particularly in machine learning and artificial intelligence, offer promising opportunities to better understand the genetic factors behind AMR emergence. It is possible to implement a machine learning framework that compares complete gene sequences from susceptible and resistant bacterial strains isolated from cattle. This approach may enable the prioritization of genes that may be involved in resistance.

## 5. Conclusions

The objective of this study was to investigate the key pathogens and antimicrobial resistance genes prevalent in foodborne pathogens isolated from cattle in the U.S. and understand their spatial distributions and time trends. By analyzing data from the NPDIB database and employing multivariate statistical analysis techniques, it was possible to uncover significant insights into the prevalence and patterns of AMR genes, pathogens, and antimicrobials with detected resistance across different states in the U.S. over time. Our findings revealed that *Salmonella* and *E.coli* were the most dominant pathogens in terms of antimicrobial resistance gene occurrences, tetracycline was the most commonly resisted antimicrobial among pathogens in cattle, and *mdsA*, *mdsB*, and *mdtM* were the most prominent genes. These results were uncovered through the principal component analysis, hierarchical clustering, projections onto the U.S. map, and a time trend analysis to provide valuable insights into the overall patterns of AMR in cattle. The maps revealed that California and Texas exhibited the highest number of pathogen cases and AMR gene detections. The time profiles demonstrated fluctuations in resistance over the years, with *E. coli* being on the rise in recent years. In summary, this study provides a comprehensive analysis of antimicrobial resistance in foodborne pathogens isolated from cattle in the U.S. This work paves the way for future study of antimicrobial resistance for different groups of cattle. The approach presented in this work can be implemented to study the antimicrobial resistance of foodborne pathogens in other farm animals.

## Figures and Tables

**Table 1:** A screenshotted portion of the data extracted from the NCBI Pathogen Detection Isolates Browser for U.S. cattle.

Organism	Collection Date	Location	Resource	50S_L22_A103V	*aac(3)-Via*	*aac(3)-IId*	Streptomycin	Sulfamethoxazole	Sulfisoxazole	Telithromycin	Tetracycline	Trimethoprim-sulfamethoxazole	Sample
4	2016	40	1	0	1	0	1	0	1	0	1	0	447
4	2017	36	1	0	0	0	1	0	0	0	0	0	450
4	2015	22	1	0	0	0	1	0	0	0	1	0	451
4	2015	22	1	0	0	0	0	0	0	0	1	0	452
4	2016	22	1	0	0	0	1	0	0	0	1	0	453
4	2015	11	1	0	0	0	0	0	0	0	0	0	535

## Data Availability

All the data used in this study can be downloaded from NCBI Pathogen Detection Isolates Browser (https://www.ncbi.nlm.nih.gov/pathogens/isolates, accessed on 15 June 2023).

## Supplementary Materials

The following supporting information can be downloaded at https://www.mdpi.com/article/10.3390/antibiotics12101509/s1, Figure S1. The projection of foodborne pathogens isolated from cattle in the U.S. onto the PC1~PC2 space; Figure S2. The projection of U.S. states base upon AMR pathogens detected in them; Figure S3. Principal component analysis performed for antimicrobials that pathogens resist in U.S. cattle; Figure S4. PCA performed to find states that had cattle that were most resistant to antimicrobials; Figure S5. Principal analysis of AMR genes in pathogens isolated from U.S. cattle; Figure S6. Principal component analysis of the states based upon cases of AMR gene detection.
