# Supplementary material for: A Comprehensive Study of Historical Detection Data for Pathogen Isolates from U.S. Cattle
Source: Antibiotics (Basel). 2023 Oct 3;12(10):1509. doi: 10.3390/antibiotics12101509 (PMC10604524; doi:10.3390/antibiotics12101509)
Supplement: Supplementary file 1 [file antibiotics-12-01509-s001.zip › antibiotics-2598466-supplementary.pdf]

Supplementary Materials

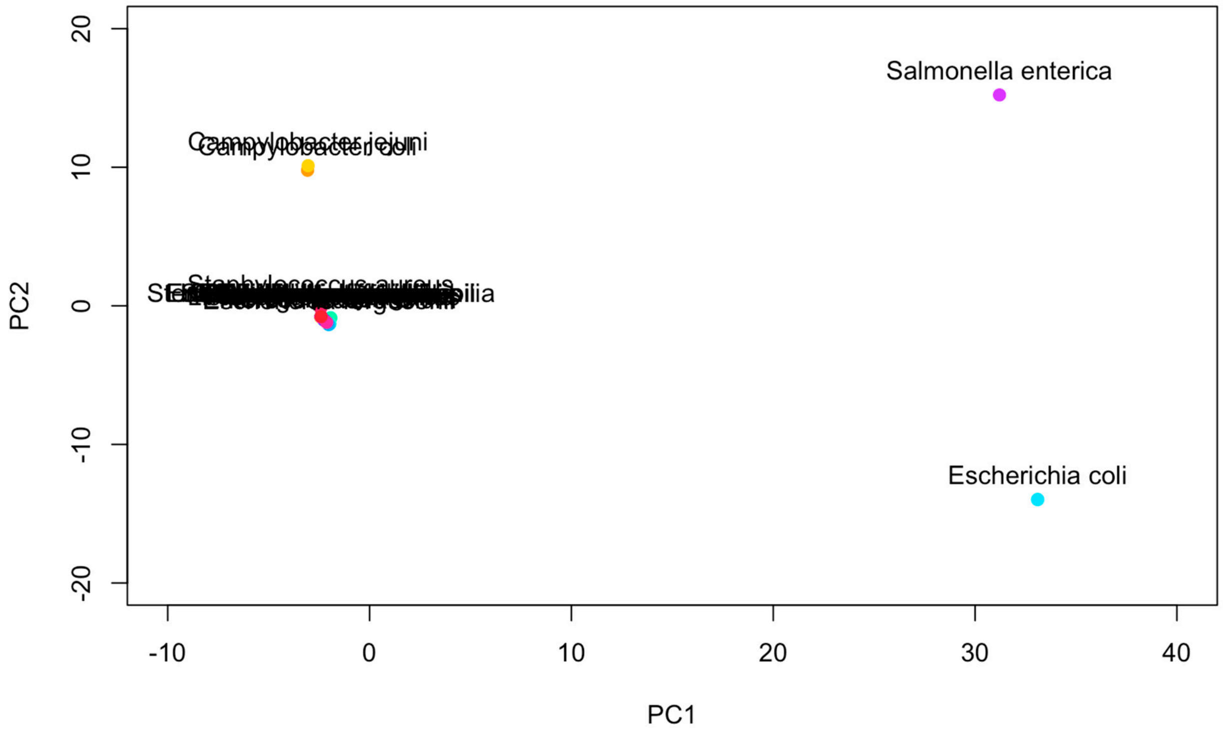

Figure S1. The projection of foodborne pathogens isolated from cattle in the U.S. onto the PC1~PC2 space.

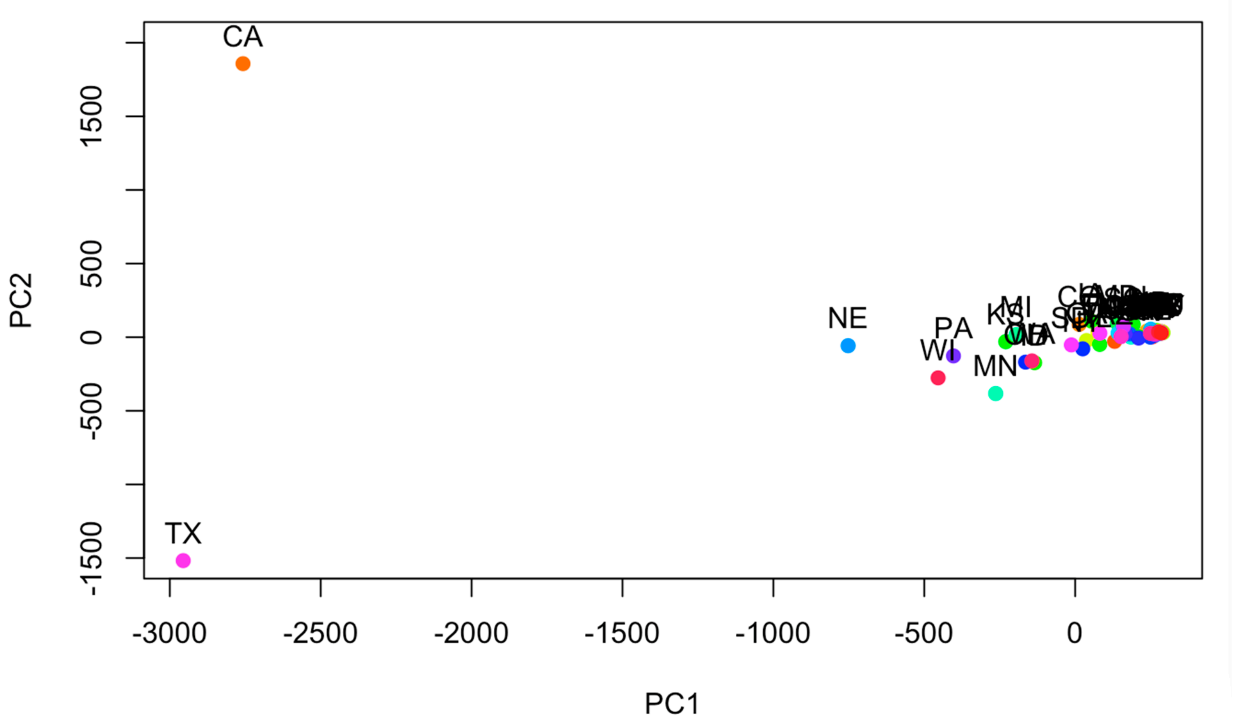

Figure S2. The projection of U.S. states base upon AMR genes detected in them.

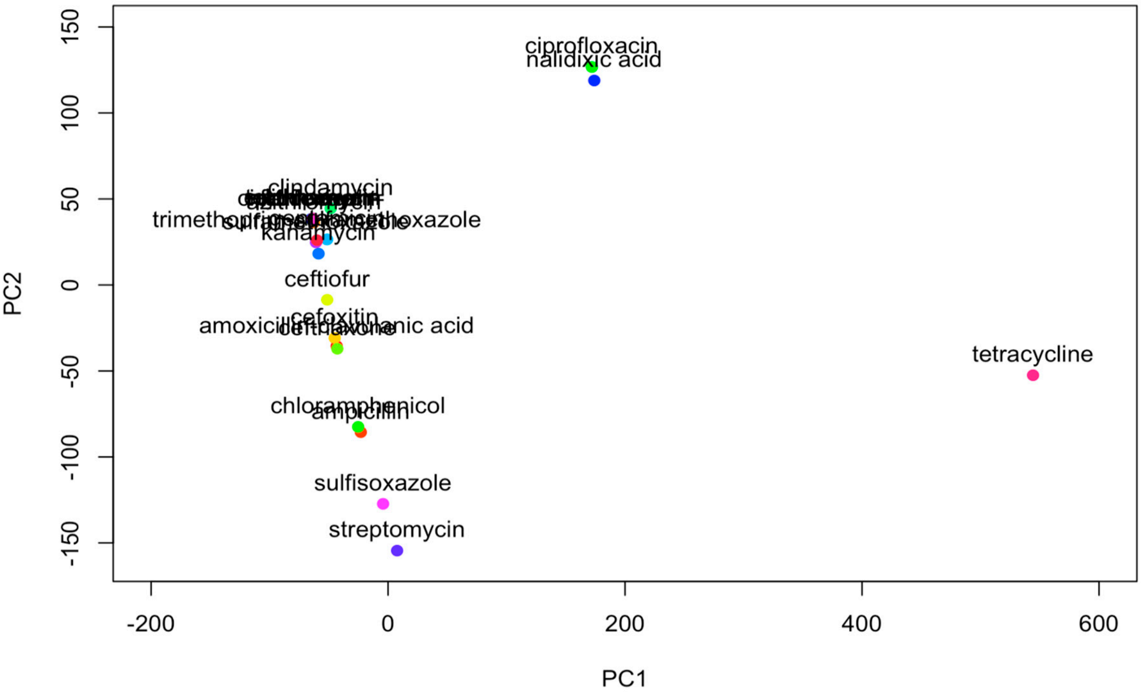

Figure S3. Principal component analysis performed for antimicrobials that pathogens resist in U.S. cattle.

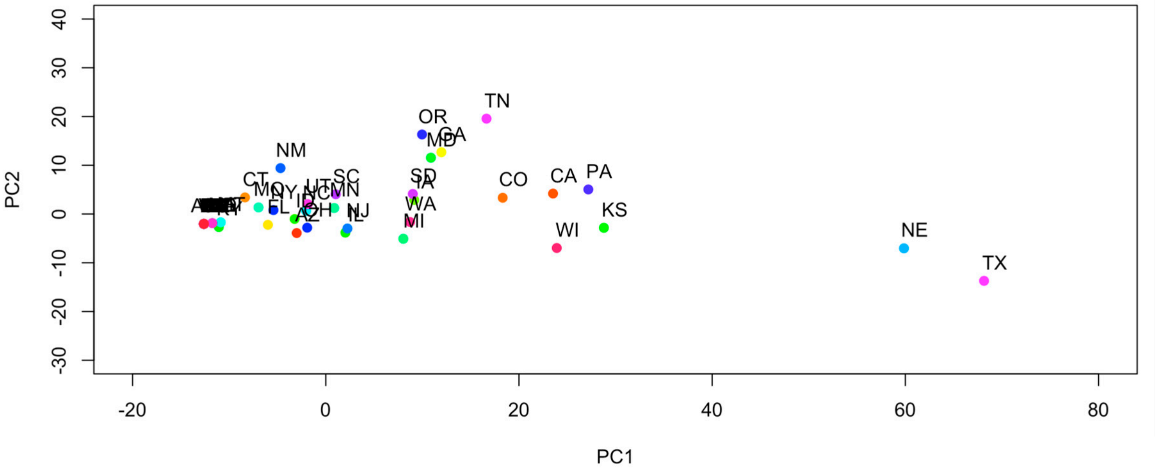

Figure S4. PCA performed to find states that had cattle that were most resistant to antimicrobials;

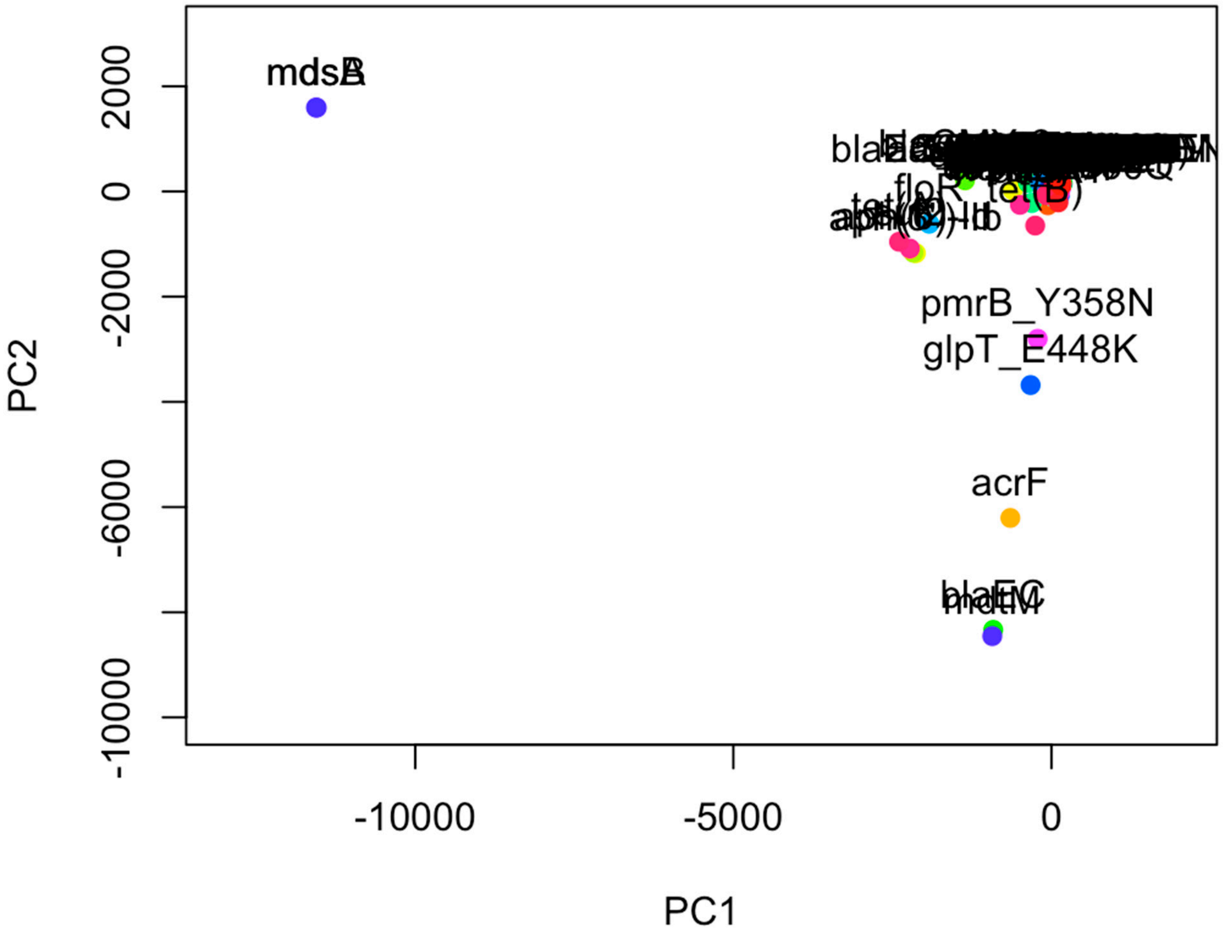

Figure S5. Principal analysis of AMR genes in pathogens isolated from U.S. cattle.

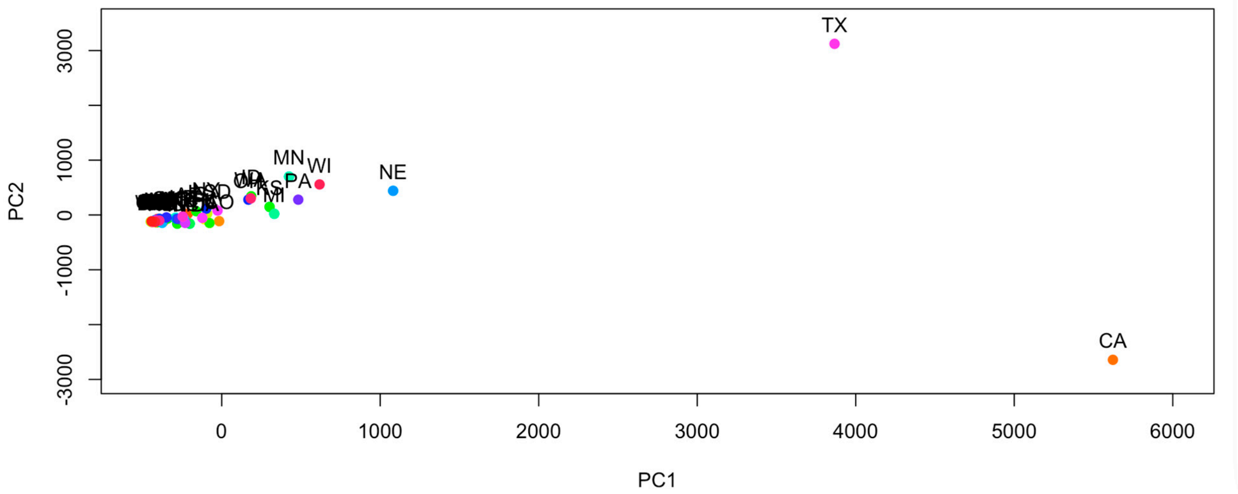

Figure S6. Principal component analysis of the states based upon cases of AMR gene detection
